# Lumbar puncture as possible cause of sudden paradoxical herniation in patient with previous decompressive craniectomy: report of two cases

**DOI:** 10.1186/s12883-017-0931-1

**Published:** 2017-08-02

**Authors:** Liang Shen, Sheng Qiu, Zhongzhou Su, Xudong Ma, Renfu Yan

**Affiliations:** 1 Department of Neurosurgery, Huzhou Central Hospital, 198 Hongqi Road, Huzhou, Zhejiang, 313000 China; 2 School of Medicine, Huzhou University, 759 East Second Ring Road, Huzhou, Zhejiang, 313000 China

**Keywords:** Case report, Lumbar puncture, Herniation, Decompressive craniectomy

## Abstract

**Background:**

Lumbar puncture is often used for the diagnosis and treatment of subarchnoid hemorrhage, infection of Cerebro-spinal Fluid (CSF), hydrocephalus in neurosurgery department patients. It is general that paradoxical herniation followed by lumbar puncture is quite rare in decompressive craniectomy cases; the related reports are very few. Moreover, most of the paradoxical herniation cases are chronic, which often occur weeks or even months after the lumbar puncture, to date, barely no reports on the acute onset paradoxical herniation have been found.

**Case presentation:**

Two traumatic brain injury patients with decompressive craniectomy (DC) and hydrocephalus suffered from a sudden paradoxical herniation after lumbar puncture. The symptoms of herniation were improved by treated with Trendelenburg position and rapid intravenous infusion.

**Conclusions:**

Lumbar puncture may have a potential risk of inducing sudden paradoxical herniation in patients with DC. CSF drainage during lumbar puncture should be in small volume for patients with DC. Once a paradoxical herniation occurs after lumbar puncture, an immediate Trendelenburg position and rapid intravenous infusion treatment may be effective.

## Background

Decompressive craniectomy (DC) is a simple yet useful emergency surgery for patients with uncontrollable intracranial pressure (ICP) to decrease ICP, especially for patients with high risks of herniation. Paradoxical herniation is a rare but life-threatening complication after DC. Paradoxical herniation is an intracranial hypotensive herniation in the direction of opposite site of the DC with subsequent brainstem compression resulting from atmospheric pressure along with brain gravity [1]. This kind of herniation with an uncommon mesencephalon compression [2] is one of the most serious sinking skin flap syndrome (SSFS). Besides, the traditional managements reducing the intracranial pressure for herniation may exacerbate paradoxical herniation, therefore, timely diagnosis and correct treatments are significantly important to combat this condition. Paradoxical herniation may also occur and become worse in patients with intracranial infection following lumbar puncture or hydrocephalus after ventriculoperitoneal shunting [3]. However, only few cases have been reported in public that paradoxical herniation may be occurred as a rare complication in patients with DC following lumbar puncture [4]. Here, we have reported and analyzed rare sudden paradoxical herniation after lumbar punctures for postoperative hydrocephalus in two traumatic brain injury (TBI) patients with a purpose to warn that lumbar puncture is a hazard in these circumstance, and once the herniation occurs it may be reversed by placing in Trendelenburg position and intravenous fluids supplements.

## Case presentation

### Patient A

**Fig.1:**
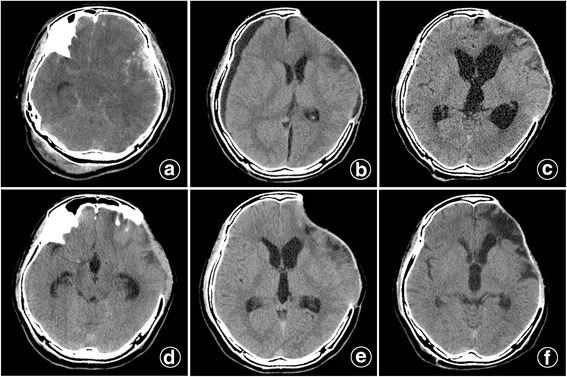
**a** A acute subdural hematoma in the left frontotemporal. **b** A repeated CT two months after the initial surgery. **c** A progressive hydrocephalus three months later. **d**, **e** Three days after placing the patient in a Trendelenburg position and giving sufficient hydration, the patient had a SSFS but no obvious midline shift. **f** Five years later, hydrocephalus improved itself without cranioplasty or ventriculoperitoneal shunt

A 29-year-old man with unconsciousness after TBI was sent and treated to our hospital in two hours after the injury, the Glasgow Coma Scale (GCS) score was used to estimate coma severity based on Eye (4), Verbal (5), and Motor (6) criteria of 5 (E1V1M3). There was a difference in diameter of pupils with 3.0 mm on the right side, and 4.0 mm on the left side; and the light reflex of eyes was disappeared. Emergency brain computed tomography (CT) revealed that patient had acute subdural hematoma in the left frontotemporal, a hemorrhagic contusion in the left frontal temporal lobe, skull fracture in the posterior occipital with epidural hematoma in the posterior fossa. Then, the patient had underwent emergent hematoma exclusion in the left frontal temporal, followed by DC. Six months later, the young man got a Glasgow Outcome Scale (GOS) of 5, Activity of Daily Living Scale (ADL) of 18, but the CT had disclosed a progressive hydrocephalus compared with three months ago. Considering the disturbance of CSF hydrodynamics, we had expected to perform cranioplasty. Before the skull repair, we conducted a lumbar puncture to release cerebrospinal fluid (CSF) and to detect and rule out the possibility of intracranial infection. Two hours later after 30 ml CSF release, the man had complained of progressively worsening dizziness and headache, and then he fell into a coma, based on the symptoms, we felt suspected paradoxical herniation might occur. Thus, the patient was placed in the Trendelenburg position and given sufficient hydration immediately. A following brain CT in three days had showed skin flap sank, but no obvious midline shift had been found. By then, the patient’s consciousness had been gradually restored, and the patient got a GCS of 15 two weeks later. The cranioplasty has not been performed for this patient in the next few years for some personal reasons. Five years later, repeated brain CT has revealed complete resolution of the midline shift, and the hydrocephalus improved itself without cranioplasty or ventriculoperitoneal shunt (Fig. 1), and the man has got a GOS of 5 and has a favorable recovery with ADL of 14.

### Patient B

**Fig. 2:**
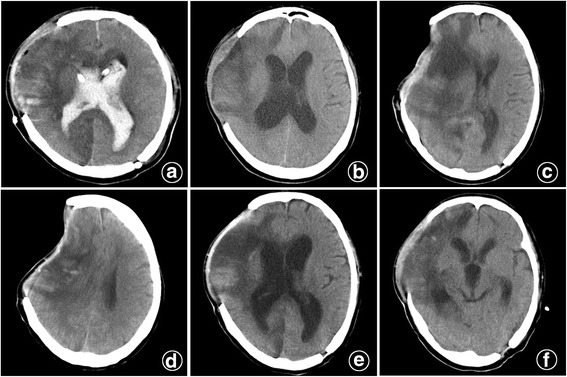
**a** A CT after the surgery of subdural hematoma in the frontal temporal lobe cleaning, craniectomy and ventricular drainage. **b** CT showed a hydrocephalus three-week later after the surgery. **c**, **d** The patient had a SSFS and midline shift after the lumbar puncture. **e**, **f** After placing the patient in a Trendelenburg positon and giving intravenous fluid, SSFS and midline shift disappeared one day later

A 56-year-old man was diagnosed with severe TBI with a GCS of 3. Pupil diameter was 4.0 mm on the right side, 2.0 mm on the left side, and pupillary reactions to light were absent. Subsequently, life-saving surgeries that cleaning subdural hematoma in the right frontal temporal lobe, DC and ventricular drainage were performed. During the course of the treatment, the patient was in a coma with a GCS of 8 (E2V1M5). The CT scanning showed a hydrocephalus in the third week after the initial surgery, accordingly, lumbar puncture was performed with an initial ICP of 200 mmHg. Subsequently, 30 ml CSF was released slowly to see immediate effects on outcome improvement. However, the man had shown some abnormal symptoms one hour later: shortness of breath, unequal size of both pupils diameters, SSFS. A Trendelenburg position and rapid saline infusion were conducted before an immediate CT scan, which showed the paradoxical herniation had occurred (Fig. 2). Two hours later, two pupil diameters were equal and pupil light reaction had become normal, the symptoms of hydrocephalus didn’t get any improvement and the consciousness disorder gradually become deepen and worsen; after many unsuccessful treatment attempts, the man was pronounced dead few weeks later due to other complications.

## Discussion

To our knowledge, most of TBI patients may have a secondary damage, which play a critical role in prognosis and has the potential to provoking death. These damage factors included subdural hematomas, intracerebral hematomas, cerebral swelling from vasospasm after subarachnoid hemorrhages, elevation of ICP. DC is generally conducted immediately in the setting of uncontrollable ICP [2]. However, TBI patients with DC may have numerous complications [5], and some of which were rare but dangerous, for example, SSFS and paradoxical herniation [4, 6–8]. The pathophysiology of paradoxical herniation has been postulated to be secondary to a large craniectomy defect exposing the intracranial contents to the external positive atmospheric pressure. This may cause also CSF disease, including post-trauma hydrocephalus. In our paper report, we have reported that two DC TBI patients with hydrocephalus, have suffered from paradoxical herniation within a few hours following lumbar puncture. The lumbar puncture may be a hazard for DC patients with hydrocephalus or intracranial infection, and it should be performed with a caution when it is necessary.

Intracranial contents exposing following craniectomy may make patients have a presentation of skin flap depression, which was known as SSFS [9]. Several decades ago, Nakamura and colleagues [10] had reported a case that performing ventriculoperitoneal shunt for hydrocephalus and cranioplasty, yet the implanted plate had to be removed due to infection in the end. It should be noted that SSFS normally occur the neurological deterioration get exacerbated, but it may reversed by making patient lying flat or in the Trendelenburg position. The paradoxical herniation risk over time may exist in this “open box” state. The proposed mechanism of paradoxical herniation may be that atmospheric pressure and the brain gravity overwhelm over the natural buoyancy that CSF provided to the brain; the brain has become collapsed after the DC, and the brain stem has get compressed [3, 4]. Doubtlessly, lumbar CSF drainage is just an effective approach in the setting of subarachnoid hemorrhage, intracranial infection. If the drainage volume is more than 200 ml/24 h, patients undergoing DC would have a high risk of SSFS [4]. Moreover, according to some reports, paradoxical herniation is a complication which occurs until days or weeks later after the lumber puncture [11, 12]. These two cases that we reported had hydrocephalus after the DC surgery; and lumbar puncture was performed to explore whether hydrocephalus had worsened the consciousness or if there were any intracranial infections. A lumbar puncture releasing slowly about 30 ml CSF leading to an acute onset paradoxical herniation within a few hours followed the drainage was observed. We believe that a rapid spontaneous CSF leak that a large volume of CSF poured out into the paraspinal soft tissues [13], was induced when conducting lumbar puncture. This proposed mechanism may explain these two cases of sudden paradoxical herniation, although we don’t have sufficient image information to support this assumption.

SSFS is a visible symptom in the process of paradoxical herniation and happen in one month to one year after DC [9]. In addition to SSFS, if patients had motor symptoms, cognitive syndrome, impaired vigilance, headaches, delay in neurological progression and other neurological symptoms, an emergent CT would be undergone generally to rule out the occurrence of paradoxical herniation [4, 9]. Considering the risk of SSFS or paradoxical herniation after lumbar puncture and shunting, neurosurgeons should be aware that cranioplasty may be the first option for DC patients with hydrocephalus. From these two cases in our department, cranioplasty was not performed before the paradoxical herniation; fortunately, timely paradoxical herniation diagnosis and appropriate treatment with Trendelenburg position and rapid intravenous infusion reverse the symptoms. This brief report is a warning, especially for young neurologists and neurosurgeons, that lumbar puncture is a hazard in DC patients. It should be emphasized that the experience of our case report are limited. More relative studies are needed to clarify the mechanism of acute onset paradoxical herniation, and the treatments for those situation warrants further confirmation and exploration.

Lumbar puncture has a risk of provoking paradoxical herniation in patients with DC [8]. Correspondingly, CSF drainage procedures need to be cautiously used in patients post craniectomy. The hypertonic saline, mannitol, hyperventilation, modest cooling, CSF drainage that lowers ICP should not be taken for the treatment of paradoxical herniation, timely diagnosis, Trendelenburg position and rapid intravenous infusion are effective measures to combat this problem. Furthermore, observing the patient undergoing lumbar puncture in case of paradoxical herniation is of vital importance.

## Conclusion

Lumbar puncture may have a potential risk of inducing sudden paradoxical herniation, and CSF drainage during lumbar puncture should be in small volume for patients with DC. Once a paradoxical herniation occurs after lumbar puncture, an immediate Trendelenburg position and rapid intravenous infusion treatment may be effective.

## Abbreviations

ADL: Activity of daily living scale
CSF: Cerebrospinal fluid
CT: Computed tomography
DC: Decompression craniectomy
GCS: Glasgow coma scale
ICP: Intracranial pressures
SSFS: Sinking skin flap syndrome
TBI: Traumatic brain injury
